# International Consensus Diagnostic Criteria for Autoimmune Pancreatitis and Its Japanese Amendment Have Improved Diagnostic Ability over Existing Criteria

**DOI:** 10.1155/2013/456965

**Published:** 2013-11-21

**Authors:** Masahiro Maruyama, Takayuki Watanabe, Keita Kanai, Takaya Oguchi, Takashi Muraki, Hideaki Hamano, Norikazu Arakura, Shigeyuki Kawa

**Affiliations:** ^1^Department of Gastroenterology, Shinshu University School of Medicine, 3-1-1 Asahi, Matsumoto 390-8621, Japan; ^2^Endoscopic Examination Center, Shinshu University School of Medicine, 3-1-1 Asahi, Matsumoto 390-8621, Japan; ^3^Center for Health, Safety, and Environmental Management, Shinshu University, 3-1-1 Asahi, Matsumoto 390-8621, Japan

## Abstract

*Objectives*. The recent International Consensus Diagnostic Criteria (ICDC) for autoimmune pancreatitis (AIP) and its Japanese amendment developed by the Japanese Pancreas Society (JPS 2011) may have overcome the drawbacks of earlier criteria and achieved a higher diagnostic ability for AIP. The aim of the present study is to evaluate this possibility and identify the underlying causes of this change. *Methods*. We compared the diagnostic abilities of the ICDC and JPS 2011 with those of the Japanese diagnostic criteria 2006 (JPS 2006), Korean diagnostic criteria (Korean), Asian diagnostic criteria (Asian), and HISORt diagnostic criteria in 110 patients with AIP and 31 patients with malignant pancreatic cancer. *Results*. The ICDC achieved the highest diagnostic ability in terms of accuracy (95.0%), followed by JPS 2011 (92.9%), Korean (92.2%), HISORt (88.7%), Asian (87.2%), and JPS 2006 (85.1%). Nearly all criteria systems exhibited a high specificity of 100%, indicating that the enhanced diagnostic ability of the ICDC and JPS 2011 likely stemmed from increased sensitivity brought about by inclusion of diagnostic items requiring no endoscopic retrograde pancreatography. The diagnostic ability of JPS 2011 was nearly equivalent to that of the ICDC. *Conclusions*. The ICDC and JPS 2011 have improved diagnostic ability as compared with earlier criteria sets because of an increase in sensitivity.

## 1. Introduction

Autoimmune pancreatitis (AIP) is a specific type of pancreatitis that is believed to be caused by autoimmune mechanisms. AIP can be classified as type 1 or type 2 based on pathological findings, for which type 1 is designated as lymphoplasmacytic sclerosing pancreatitis (LPSP) and type 2 is termed idiopathic duct-centric chronic pancreatitis (IDCP) or AIP with granulocytic epithelial lesion (GEL).

Over the last decade, many diagnostic criteria for AIP have been proposed and revised under various circumstances. In 2002, the first set of criteria for AIP diagnosis was established by the Japanese Pancreatic Society (JPS) (JPS 2002) [1, 2], which was subsequently revised in 2006 (JPS 2006) by the JPS and the nationally supported Research Committee of Intractable Diseases of the Pancreas [3]. JPS 2002 and JPS 2006 consisted of three main items: characteristic imaging findings, serology, and pathological findings. Both systems incorporated endoscopic retrograde pancreatocholangiography (ERCP) in imaging findings, and IgG4 was included in serological evaluation in JPS 2006. In the same period of the JPS 2006 proposal, the HISORt [4] and Korean diagnostic criteria [5] were released by Chari et al. of the Mayo Clinic in the United States and Kim et al. of the Asan Medical Center in Korea, respectively. Pathological findings were a major item in the HISORt criteria, and other organ involvement (OOI) and response to steroid (Rt) were newly introduced as diagnostic parameters. ERCP in imaging findings was not considered to be a major factor. The Korean diagnostic criteria were revised by the Korean Biliary Tract Pancreas Society in 2007 [6, 7]. This version's structure was virtually identical to those of JPS 2002 and JPS 2006, except that it further distinguished definitive from probable diagnosis and added the diagnostic item of Rt. In the Japanese criteria, Rt had not been adopted because it was considered to increase the risk of an incorrect diagnosis of pancreatic cancer as AIP, resulting in a delay in surgical resection. In 2008, the Asian diagnostic criteria [8] were proposed as a collaborative work between Japanese and Korean researchers, whereby AIP diagnosis could be made using characteristic pathological findings of the pancreas only. Rt was included as an option if evaluated at a professional institution after excluding the possibility of pancreatic cancer. The Mayo Clinic modified the HISORt criteria in 2009 on the basis of a comparative study of AIP and pancreatic cancer [9].

Subsequent to these events, the need for uniform international diagnostic criteria that integrated all of the various existing systems became evident [10, 11]. Several symposia to discuss the issue were held since 2009 by global pancreatology experts, which were cumulated in the International Consensus Diagnostic Criteria (ICDC) for AIP [12] in 2011. The ICDC enabled the diagnosis of AIP based on an international standard and defined type 1 and type 2 AIP according to pathological and clinical findings with a view to clarify the clinical features, pathogenesis, and natural history of the disease. However, because the ICDC were intended for experts of pancreatology, they were often difficult to employ for general internists. Therefore in Japan, the revised JPS 2011 diagnostic criteria [13] were released; they were compatible with the ICDC and familiar to general internists and included country-specific parameters. The major difference between the ICDC and JPS 2011 was that the Japanese criteria focused on type 1 AIP and required evaluation with endoscopic retrograde pancreatography (ERP) in indeterminate imaging evidence [13, 14].

The extensive revision process undertaken on the diagnostic criteria for AIP by the ICDC and JPS 2011 was thought to overcome the various drawbacks of existing criteria and improve diagnostic ability. The key features of the 6 sets of diagnostic criteria evaluated in this report are shown in Table 1 [11]. The aim of the present study was to determine whether the diagnostic ability of the ICDC and JPS 2011 has actually improved. We also sought to identify what factors may have contributed to this change, whether the new diagnostic criteria can sufficiently exclude pancreatic cancer, and what new problems have appeared in comparison with the earlier JPS 2006, Korean, Asian, and HISORt criteria.

## 2. Materials and Methods

### 2.1. Study Subjects

We enrolled 110 Japanese AIP patients who were treated between August 1992 and January 2013 at Shinshu University Hospital and reevaluated them using each of the following 6 sets of diagnostic criteria: JPS 2006, Korean, Asian, HISORt, ICDC, and JPS 2011. Our cohort consisted of 84 men and 26 women whose median age (range) was 66 (38–85) years. Since we focused on clinical studies of type 1 AIP, we considered AIP to be type 1 AIP in this report.

AIP diagnosis was performed based on previously available diagnostic criteria in Japan, namely, JPS 2002 from 2002, JPS 2006 from 2006, Asian from 2008, and JPS 2011 or ICDC from 2011. Prior to 2002, AIP diagnosis was based on our own private diagnostic criteria that included imaging findings of irregular narrowing of the main pancreatic duct and sonolucent swelling of the pancreas that responded to glucocorticoid treatment, as well as serology findings of obstructive jaundice, hypergammaglobulinemia, and high serum IgG concentrations [15], all of which were proposed by Yoshida et al. in 1995 [16] but later reevaluated by JPS 2002.

As a malignant disorder control group, we enrolled 31 patients who exhibited pancreatic mass lesion and were ultimately diagnosed as having malignant pancreatic cancer that was confirmed pathologically after surgery. Their serum IgG4 values were recorded since they were suspected of having AIP. Twenty patients were men and 11 were women, and median age (range) was 66 (27–83) years. The final pathological diagnosis of patients in the control group was invasive ductal carcinoma (28 patients), neuroendocrine neoplasm (2 patients), and intraductal papillary-mucinous neoplasm (1 patient).

### 2.2. Methods

We examined the sensitivity, specificity, and accuracy of all 6 sets of diagnostic criteria for AIP and then compared the ICDC with the earlier criteria. After identifying the AIP patients who were newly detected by the ICDC, we examined for underlying factors that may have contributed to an improvement in diagnostic ability. Next, we selected 33 AIP patients from our cohort (25 men and 8 women; median age (range): 66 (27–83) years) with focal or segmental pancreatic swelling defined as level 2 parenchymal imaging by the ICDC that could be difficult to discriminate from malignant pancreatic tumors. The sensitivity, specificity, and accuracy of all 6 sets of diagnostic criteria were measured for these patients. Lastly, we examined in the same manner as the ICDC if JPS 2011 also had improved diagnostic ability for AIP and whether it had a comparable diagnostic ability to the ICDC.

## 3. Results

### 3.1. Comparison of Diagnostic Abilities of 6 Sets of Diagnostic Criteria for AIP

The sensitivity, specificity, and accuracy of each diagnostic system are presented in Table 2. Diagnostic accuracy for definite AIP was highest for the ICDC (95.0%), followed by JPS 2011 (92.9%), Korean (92.2%), HISORt (88.7%), Asian (87.2%), and JPS 2006 (85.1%), which showed that the accuracy of both the ICDC and JPS 2011 surpassed that of the earlier 4 sets of criteria. With regard to specificity, all 6 criteria systems achieved perfect or near-perfect results, implying that the reason for the improved diagnostic ability of the ICDC and JPS 2011 was increased sensitivity.

### 3.2. Factors Contributing to a Revised ICDC Diagnosis

The number of patients who could not be diagnosed by the earlier 4 sets of criteria was 21 for JPS 2006, 10 for Korean (not definitive cases), 18 for Asian, and 16 for HISORt. The factors contributing to a revised diagnosis by the ICDC are shown in Table 3. The ICDC could diagnose most cases missed by the other sets of criteria, which confirmed their higher sensitivity and accuracy.

#### 3.2.1. ICDC versus JPS 2006

Nineteen of 21 patients (90%) who were negative for definitive AIP according to JPS 2006 could be identified by the ICDC. The contributing factors were histology in 3 patients whose imaging evidence was indeterminate; combination of typical imaging evidence and collateral evidence, such as serology and/or OOI, in 12 patients; and combination of indeterminate imaging evidence and collateral evidence for which all evidence other than ERP was required to be level 1 in 4 patients. Thus, imaging evidence not requiring ERCP greatly contributed to diagnostic improvement. No additional cases of AIP could be diagnosed by the factor of Rt according to the ICDC algorithm. There were 2 patients with AIP-not otherwise specified who might have had type 2 AIP as classified by the ICDC.

#### 3.2.2. ICDC versus Korean Criteria

All 10 patients (100%) who were negative for definitive AIP according to the Korean criteria were identified by the ICDC. The contributing factors were similar to those for JPS 2006, namely, histology and combination of typical imaging evidence and collateral evidence in 3 patients each and combination of indeterminate imaging evidence and collateral evidence in 4 patients. As with JPS 2006, imaging evidence not requiring ERCP greatly contributed to diagnostic improvement, and again Rt was not a factor in revised AIP diagnosis by the ICDC algorithm.

#### 3.2.3. ICDC versus Asian Criteria

Sixteen of 18 patients (89%) who were negative for definitive AIP according to the Asian criteria could be identified by the ICDC. With the exception of histology, the contributing factors were also similar to those for JPS 2006 as combination of typical imaging evidence and collateral evidence in 12 patients and combination of indeterminate imaging evidence and collateral evidence in 4 patients. There were 2 patients with AIP-not otherwise specified who might have had type 2 AIP according to the ICDC. The benefit of no ERCP requirement and the absent role of Rt in new diagnosis were evident in this group as well.

#### 3.2.4. ICDC versus HISORt

Eleven of 16 patients (69%) who were negative for definitive AIP according to HISORt were identified by the ICDC. The contributing factors were combination of typical imaging evidence and collateral evidence in 5 patients and combination of indeterminate imaging evidence and collateral evidence in 6 patients. There were 4 patients with AIP-not otherwise specified who might have had type 2 AIP as classified by the ICDC and 1 patient who remained undiagnosed.

### 3.3. Cases of AIP Undiagnosable by the ICDC

There was only 1 AIP case that could be diagnosed by the JPS 2006, Korean, and Asian criteria but not by the ICDC. This patient was identified using a combination of indeterminate imaging, ERP, and serum autoantibody; no signs related to histology, serology, or OOI were evident, which resulted in a false negative diagnosis by the ICDC. There were no such cases encountered with the HISORt criteria.

### 3.4. Factors Contributing to a Revised JPS 2011 Diagnosis

JPS 2011 could also newly diagnose many AIP cases that were missed by the earlier sets of criteria. Among the 21, 10, 18, and 16 patients who were negative for definitive AIP using the JPS 2006, Korean, Asian, and HISORt criteria, 15 (71%), 6 (60%), 12 (67%), and 8 patients (50%), respectively, were identified as having definitive AIP by JPS 2011. The contributing factors to the revised diagnoses were comparable to those of the ICDC (Table 4).

### 3.5. Comparison of the Diagnostic Abilities of the 6 Sets of Diagnostic Criteria for Focal/Segmental AIP

As AIP patients with focal or segmental pancreatic swelling may be difficult to discriminate from those with pancreatic malignancy, we evaluated whether the ICDC and JPS 2011 could improve diagnostic ability in this area as well. The sensitivity, specificity, and accuracy of all 6 sets of diagnostic criteria for this form of AIP are listed in Table 5. Diagnostic accuracy for definite AIP was highest for the ICDC (95.3%), followed by Asian (93.8%), JPS 2006 (90.6%) and JPS 2011 (90.6%), Korean (89.1%), and HISORt (89.1%). Again, the accuracy of the ICDC surpassed those of the earlier 4 sets of criteria. JPS 2011, for which ERP is an essential diagnostic factor in such patients, did not display the improvement of diagnostic ability seen for the ICDC.

### 3.6. Comparison of ICDC and JPS 2011 Diagnostic Abilities

Lastly, we compared the diagnostic abilities of the ICDC and JPS 2011, focusing especially on mismatched cases. Among our 110 AIP patients, 103 patients (93.6%) had a matching diagnosis, which consisted of 98 patients (89.1%) with a definitive diagnosis, 4 patients (3.6%) with a possible diagnosis, and 1 patient (0.9%) with no diagnosis. The remaining 7 patients (6.4%) whose diagnoses did not match are presented in Table 6. The major factor accounting for a discrepancy was combination of indeterminate imaging evidence and collateral evidence of L1 serology and L1 OOI; whereas the ICDC made a definitive diagnosis, JPS 2011 did not due to the absence of ERP.

## 4. Discussion

### 4.1. Were the Diagnostic Abilities of the ICDC and JPS 2011 Improved?

The present study revealed the following results: (1) both the ICDC and JPS 2011 had improved diagnostic ability for AIP in terms of accuracy as compared with 4 earlier diagnostic systems; (2) the specificity of all criteria sets was comparably high at 100% or nearly 100%, so all could reliably differentiate between AIP and pancreatic cancer; and (3) based on the high specificity of all criteria systems, the improvement in diagnostic ability seen for the ICDC and JPS 2011 likely stemmed from an elevation in sensitivity, which accounted for virtually all of the cases newly diagnosed by these 2 diagnostic systems.

This study confirms a previous smaller report that showed the sensitivities of 5 major criteria sets to be 95.1% (ICDC), 90.2% (Korean), 86.9% (JPS 2011), and 83.6% (Asian and HISORt) [17]. A high specificity for all systems was also noted [17].With regard to AIP cases with focal or segmental pancreatic swelling, especially those difficult to discriminate from malignant pancreatic tumor [18–20], the diagnostic ability of JPS 2011 trailed that of the ICDC due to its requirement for ERP evaluation.

### 4.2. Main Factors Contributing to a Revised Diagnosis Using the ICDC and JPS 2011

The major factors that enabled the ICDC and JPS 2011 to identify AIP patients who had been missed by the earlier 4 criteria sets were diagnostic items requiring no ERP, such as histology and the combination of typical imaging evidence and the collateral evidence of serology or OOI. Thus, in the case of all former criteria systems except for HISORt, diagnostic evidence not requiring ERP greatly contributed to diagnostic improvement. JPS 2011 requires evaluation with ERP in indeterminate imaging evidence to avoid a misdiagnosis of pancreatic cancer, and so its sensitivity was slightly lower than that of the ICDC. Indeed, although several ERP findings are useful for differentiating AIP from pancreatic cancer [21, 22], the ability to diagnose AIP with ERP alone is limited. A previous study showed that the diagnostic sensitivity for segmental/focal type AIP may increase with the combination of CT and ERP [17].

No new cases of definite AIP were diagnosed by the factor of Rt using the ICDC algorithm. Therefore, Rt as a diagnostic item appears to be of lesser value for AIP and furthermore may delay surgery for patients with pancreatic cancer. It is possible, however, that the role of Rt may have been underestimated due to selection bias in this study because AIP was determined according to previous Japanese diagnostic criteria in which the disease was diagnosed without steroid responsiveness. On this subject, an earlier study recommended a diagnostic algorithm using CT for diffuse type AIP and a combination of CT and ERP followed by EUS-FNA for segmental/focal type AIP [17], while other reports have advocated a short trial period of steroid therapy for differentiation between AIP and pancreatic cancer [5, 23]. Lastly, as there was only 1 case of AIP that was diagnosed by the earlier 4 criteria sets but not by the ICDC or JPS 2011, it may be possible to disregard newly negative cases indicated by these 2 systems.

### 4.3. Overcoming the Shortcomings of the ICDC

Although we observed that the ICDC improved diagnostic ability as compared with earlier diagnostic criteria for AIP, the ICDC are complicated and often difficult to use for general internists because they are intended for pancreatology experts. In addition, since the ICDC were designed to be used globally, several country-specific diagnostic criteria that were particularly suited to local diagnostic conditions might have been excluded in the process. The ICDC also divide criteria into level 1 (L1) and level 2 (L2) to improve diagnostic ability, especially in the differentiation between AIP and pancreatic cancer, but this distinction appears to complicate diagnosis. Sumimoto et al. validated the classification of L1/L2 in parenchymal and ductal findings, IgG4 and OOI, although they stated that it seemed unnecessary that IgG4 and OOI be categorized as L1 or L2 [17]. Therefore, it may be of benefit to establish diagnostic criteria specific to individual countries that are not only compatible with the ICDC but are also convenient and popular among doctors. In Japan, JPS 2011 was proposed to be compatible with the ICDC while enabling general internists to diagnose AIP patients as accurately as with the international standard. JPS 2011 contains independent pancreas imaging of parenchyma and ducts, a single serology of IgG4, OOI, and an optional steroid trial for type 1 AIP that are not distinguished as level 1/2, which appears to maintain agreement with diagnosis using the ICDC [17]. We found the sensitivity of JPS 2011 was slightly lower than that of the ICDC due to its requirement for ERP evaluation of indeterminate imaging evidence. It can be said that a great deal of emphasis is placed on the discrimination between AIP and pancreatic cancer among Japanese doctors [21, 22], thus making ERP an important requirement for uniform national diagnostic criteria. A previous study also demonstrated comparable results between JPS 2011 and the ICDC [17]. Similarly to JPS 2011, other countries may need to establish adjusted diagnostic criteria that are convenient and compatible with the ICDC and include local diagnostic requirements.

### 4.4. Limitations of the Present Study

The present study has limitations inherent to its retrospective nature. In addition, AIP in our patient cohort had been diagnosed using different diagnostic criteria sets, so selection bias may have affected the results.

### 4.5. Conclusion and Future Prospective

In this report, the ICDC and JPS 2011 showed improved diagnostic ability for AIP compared with former criteria as a result of increased sensitivity. The major contributing factors for this amelioration were diagnostic items requiring no ERP tests, such as histology and combination of typical imaging evidence with the collateral evidence of serology and OOI. As we could not evaluate the diagnostic ability for type 2 AIP in the current study, further research is needed in this area; there were several cases of AIP-not otherwise identified using the ICDC that might have been type 2 AIP. Multinational, multicenter studies on the applicability of country-specific adaptations of the ICDC are advised.

## Figures and Tables

**Table 1:** Comparison of 6 major diagnostic criteria systems for AIP.

	JPS-2006	Korean	Asian	HISORt		ICDC		JPS-2011	
(I) Imaging findings	Mandatory	Mandatory	Mandatory	Not mandatory		Not mandatory		Not mandatory	
				Typical	Atypical	Typical	Indeterminate	Typical	Indeterminate

(a) Parenchymal imaging	Swelling	Swelling	Swelling	Swelling	Focal mass/ calcification/ atrophy, etc.	Diffuse swelling	Segmental/ focal swelling	Diffuse swelling	Segmental/ focal swelling

(b) Ductal imaging	ERCP irregular narrowing	ERCP/MRCP irregular narrowing	ERCP irregular narrowing	Irregular narrowing	Focal duct stricture	Irregular narrowing			ERCP^¶^ Irregular narrowing

(II) Serology	*γ*-Globulin/ IgG/IgG4/ autoantibodies	IgG/IgG4/ autoantibodies	IgG/IgG4/ autoantibodies	IgG4		IgG4		IgG4	

(III) Histology	LPSP	LPSP/IgG4-positive plasma cells	LPSP/IgG4-positive plasma cells	LPSP/IgG4-positive plasma cells		LPSP/IgG4-positive plasma cells IDCP/GEL^†^		LPSP/IgG4-positive plasma cells	

(IV) Other organ involvement	Not included	Includes histological findings	Not included	Includes histological findings		Includes histological findings/ clinical findings^Φ^		Includes histological findings/ clinical findings	

(V) Response to steroid	Not included	Includes pancreatic lesion/ extrapancreatic lesions	Optional pancreatic lesion	Includes pancreatic lesion/ extra-pancreatic lesions		Includes pancreatic lesion/ extra-pancreatic lesions		Includes pancreatic lesion/ extra-pancreatic lesions	

Diagnosis	I + II I + III	I + II I + III I + IV I + V	I + II I + III	III I (typical) + II I (atypical) + II/IV + V		Shown in Table 3		Shown in Table 4	

^¶^JPS-2011 requires evaluation by ERP in indeterminate imaging evidence.

^†^Diagnosis of type 2 AIP requires histologically confirmed IDCP/GEL.

^Φ^Findings in type 1 AIP are sclerosing cholangitis, retroperitoneal fibrosis, Mikulicz disease, and renal disease. Finding in type 2 AIP is inflammatory bowel disease.

ERCP: endoscopic retrograde pancreatocholangiography; LPSP: lymphoplasmacytic sclerosing pancreatitis; IDCP: idiopathic duct-centric chronic pancreatitis; GEL: granulocytic epithelial lesion.

**Table 2:** Sensitivity, specificity, and accuracy of each diagnostic criteria system for AIP.

	JPS-2006	Korean	Asian	HISORt	ICDC	JPS-2011
Sensitivity						
Definitive	80.9% (89/110)	90.9% (100/110)	83.6% (92/110)	85.4% (94/110)	93.6% (103/110)	90.9% (100/110)
Probable		98.2% (108/110)			95.5% (105/110)	92.7% (102/110)

Specificity	100% (31/31)	96.8% (30/31)	100% (31/31)	100% (31/31)	100% (31/31)	100% (31/31)

Accuracy						
Definitive	85.1% (120/141)	92.2% (130/141)	87.2% (123/141)	88.7% (125/141)	95.0% (134/141)	92.9% (131/141)
Probable		97.9% (138/141)			96.5% (136/141)	94.3% (133/141)

**Table 3:** ICDC diagnosis of negative AIP cases determined by earlier sets of criteria.

ICDC				JPS-2006	Korean	Asian	HISORt
				No diagnosis	Not definitive	No diagnosis	No diagnosis
Number				21	10	18	16

Diagnosis	Primary basis	Imaging evidence	Collateral evidence				

	Histology	Typical/indeterminate	L1 histology	3	3		

			L1/L2 serology	2		2	
	Imaging	Typical	L1/L2 OOI	4	1	4	3
			L1/L2 serology/OOI	6	2	6	2
			L2 histology				

			L1 serology + L1 OOI	3	3	3	2
(1) Definitive type 1 AIP		Indeterminate	L2 ERP + L1 serology				2
			L2 ERP + L1 OOI	1	1	1	2

			L1 serology + Rt				
			L1 OOI + Rt				
	Response to steroid	Indeterminate	L1 ERP + L2 serology + Rt				
			L1 ERP + L2 OOI + Rt				
			L1 ERP + L2 histology + Rt				

			L2 serology + Rt				
(2) Probable type 1 AIP		Indeterminate	L2 OOI + Rt				
			L2 histology + Rt				

(3) AIP-not otherwise specified		Typical	L1/2 ERP + Rt	2		2	4
		Indeterminate	L1/2 ERP + Rt				

(4) No diagnosis							1

L1 and 2: levels 1 and 2; OOI: other organ involvement; ERP: endoscopic retrograde pancreatography; Rt: response to steroid.

**Table 4:** JPS-2011 diagnosis of negative AIP cases determined by earlier sets of criteria.

JPS-2011				JPS-2006	Korean	Asian	HISORt
				No diagnosis	Not definitive	No diagnosis	No diagnosis
Number				21	10	18	16

Diagnosis	Primary basis	Imaging evidence	Collateral evidence				

	Histology	Typical/indeterminate	Histology	3	3		

			Serology	2		2	
	Imaging	Typical	OOI	4	1	4	3
			Serology/OOI	6	2	6	2
			Histology				

			ERP + serology + OOI				2
(1) Definitive AIP		Indeterminate	ERP + serology + histology				
			ERP + OOI + histology				

			ERP + serology + Rt				
	Response to steroid	Indeterminate	ERP + OOI + Rt				1
			ERP + histology + Rt				

			ERP + serology				1
(2) Probable AIP		Indeterminate	ERP + OOI	1	1	1	1
			ERP + histology				

(3) Possible AIP		Typical	ERP + Rt	2		2	4
		Indeterminate	ERP + Rt				

(4) No diagnosis				3	3	3	2

OOI: other organ involvement; ERP: endoscopic retrograde pancreatography; Rt: response to steroid.

**Table 5:** Sensitivity, specificity, and accuracy of each diagnostic criteria system for indeterminate AIP.

	JPS-2006	Korean	Asian	HISORt	ICDC	JPS-2011
Sensitivity						
Definitive	81.8% (27/33)	81.8% (27/33)	87.9% (29/33)	78.8% (26/33)	90.9% (30/33)	81.8% (27/33)
Probable		97.0% (32/33)			97.0% (32/33)	87.9% (29/33)

Specificity	100% (31/31)	96.8% (30/31)	100% (31/31)	100% (31/31)	100% (31/31)	100% (31/31)

Accuracy						
Definitive	90.6% (58/64)	89.1% (57/64)	93.8% (60/64)	89.1% (57/64)	95.3% (61/64)	90.6% (58/64)
Probable		96.9% (62/64)			98.4% (63/64)	93.8% (60/64)

**Table 6:** Comparison of diagnostic abilities of the ICDC and JPS-2011 related to mismatched cases.

ICDC				JPS-2011			
				Definitive	Probable	Possible	No diagnosis
Number				2	2		3

Diagnosis	Primary basis	Imaging evidence	Collateral evidence				

	Histology	Typical/indeterminate	L1 histology				

			L1/L2 serology				
	Imaging	Typical	L1/L2 OOI				
			L1/L2 serology/OOI				
			L2 histology				

			L1 serology + L1 OOI				3
(1) Definitive type 1 AIP		Indeterminate	L2 ERP + L1 serology		1		
			L2 ERP + L1 OOI		1		

			L1 serology + Rt				
			L1 OOI + Rt				
	Response to steroid	Indeterminate	L1 ERP + L2 serology + Rt				
			L1 ERP + L2 OOI + Rt				
			L1 ERP + L2 histology + Rt				

			L2 serology + Rt	1			
(2) Probable type 1 AIP		Indeterminate	L2 OOI + Rt				
			L2 serology/L2 OOI + Rt	1			
			L2 histology + Rt				

(3) AIP-not otherwise specified		Typical	L1/2 ERP + Rt				
		Indeterminate	L1/2 ERP + Rt				

(4) No diagnosis							

L1 and 2: levels 1 and 2; OOI: other organ involvement; ERP: endoscopic retrograde pancreatography; Rt: response to steroid.
